# The rule of declining adaptability in microbial evolution experiments

**DOI:** 10.3389/fgene.2015.00099

**Published:** 2015-03-11

**Authors:** Alejandro Couce, Olivier A. Tenaillon

**Affiliations:** Unité Mixte de Recherche 1137 (IAME-INSERM) Paris, France

**Keywords:** beneficial mutations, distribution of fitness effects, epistasis, finite-sites model, Fisher's model

## Abstract

One of the most recurrent observations after two decades of microbial evolution experiments regards the dynamics of fitness change. In a given environment, low-fitness genotypes are recurrently observed to adapt faster than their more fit counterparts. Since adaptation is the main macroscopic outcome of Darwinian evolution, studying its patterns of change could potentially provide insight into key issues of evolutionary theory, from fixation dynamics to the genetic architecture of organisms. Here, we re-analyze several published datasets from experimental evolution with microbes and show that, despite large differences in the origin of the data, a pattern of inverse dependence of adaptability with fitness clearly emerges. In quantitative terms, it is remarkable to observe little if any degree of idiosyncrasy across systems as diverse as virus, bacteria and yeast. The universality of this phenomenon suggests that its emergence might be understood from general principles, giving rise to the exciting prospect that evolution might be statistically predictable at the macroscopic level. We discuss these possibilities in the light of the various theories of adaptation that have been proposed and delineate future directions of research.

## Introduction

The use of microbes to experimentally address evolutionary questions blossomed after molecular biology tools became routine two decades ago (Lenski and Travisano, 1994; Elena and Lenski, 2003). Since then, the literature on microbial experimental evolution has increased substantially, virtually covering the entire range of themes of the Modern Synthesis (Buckling et al., 2009). Regardless of the particular question at hand, the general result is that populations increase their level of adaptation over time; which is usually reported as some metric of the growth rate of the progeny relative to the ancestor (Chevin, 2011). Adaptation is, by definition, the main macroscopic outcome of Darwinian evolution. Therefore, the patterns of adaptive change could potentially inform about key issues of evolutionary theory, from fixation dynamics to the genetic architecture of organisms.

A recurrent pattern that emerges from a variety of experimental systems is that the rate of change in fitness over time (i.e., rate of adaptation) depends on the current level of adaptation of a genotype. This phenomenon is observed in two types of studies. In the first, the archetypal evolution experiment, populations derived from the same ancestor are serially propagated in a new environment. After an initial phase of rapid adaptation, fitness improvement typically decelerates (Wiser et al., 2013; Good and Desai, 2015). The second type of experiments directly assesses the dependence of the rate of adaptation on initial fitness. A collection of genotypes with different fitness values are allowed to evolve during some time period, and thereafter their fitness increase is compared to that of the reference strain. Although there is some variability in the results, the overall trend is that the most unfit genotypes tend to display the fastest rates of adaptation (Moore et al., 2000; Sanjuan et al., 2005; Barrick et al., 2010; Kryazhimskiy et al., 2014).

Here, we aim to review this general pattern and its implications for evolutionary theory. To gain insight into its nature, we performed a comparative analysis of published datasets from experimental evolution in a variety of species. Despite considerable differences in the origin of the data, all of them share a universal property: the approximate log-log linear dependence of the rate of adaptation with initial fitness. This regularity suggests that general principles may be found across diverse evolving organisms, pointing to the intriguing prospect that evolution might perhaps be predictable at the macroscopic level (Good and Desai, 2015). We discuss these possibilities in the light of the various theories of adaptation that have been proposed in the literature.

## Patterns of declining adaptability

The first reports on the proportionality of the rate of adaptation and initial fitness originated in the context of compensatory evolution. The original question was to what degree populations that had accumulated deleterious mutations, (e.g., costly phage or antibiotic resistances) were capable of recovering fitness within the relevant evolutionary timescale (Lenski, 1988; Burch and Chao, 1999; Moore et al., 2000; Reynolds, 2000; Maisnier-Patin et al., 2002). Although examples of declining adaptability are common in this field (Moore et al., 2000; Estes and Lynch, 2003; Sanjuan et al., 2005; Moore and Woods, 2006; Gifford et al., 2011; Sousa et al., 2012), we chose not to include them in this work mainly due to the small sample size of most studies. Our analysis was instead restricted to five recent studies that explicitly evaluated the rate of adaptation as a function of initial fitness.

The five studies considered here cover a range of species that includes eukaryotes, prokaryotes and virus; yet their general experimental design is essentially the same: a collection of strains with different starting fitness are evolved in a defined laboratory environment, and after hundreds of generations, their fitness gains are evaluated. The method employed for assembling each collection is, however, different in every case. In Szamecz et al. (2014), 187 mutants with various growth rate defects were selected from a publicly available collection of single-gene deletion strains of *Saccharomyces cerevisiae*. Kryazhimskiy et al. (2014), also working with *S. cerevisae*, allowed 64 parallel lines founded from the same strain to adaptively diverge for 240 generations prior to the onset of the experiment. Crucially, they measured fitness improvements at two different timepoints, which are independently included in our analysis. Barrick et al. (2010) selected 8 point mutation mutants of *Escherichia coli* resistant to the antibiotic rifampicin, a well-known model of costly antibiotic resistance. In Perfeito et al. (2014), 23 independent lineages from a hypermutable *E. coli* strain were propagated through severe bottlenecks for >20 cycles, resulting in an intense accumulation of deleterious mutations. Rokyta et al. (2009) employed 8 different microviridae strains, chosen according to their diverse infection efficiency in an *E. coli* host.

**Figure 1:**
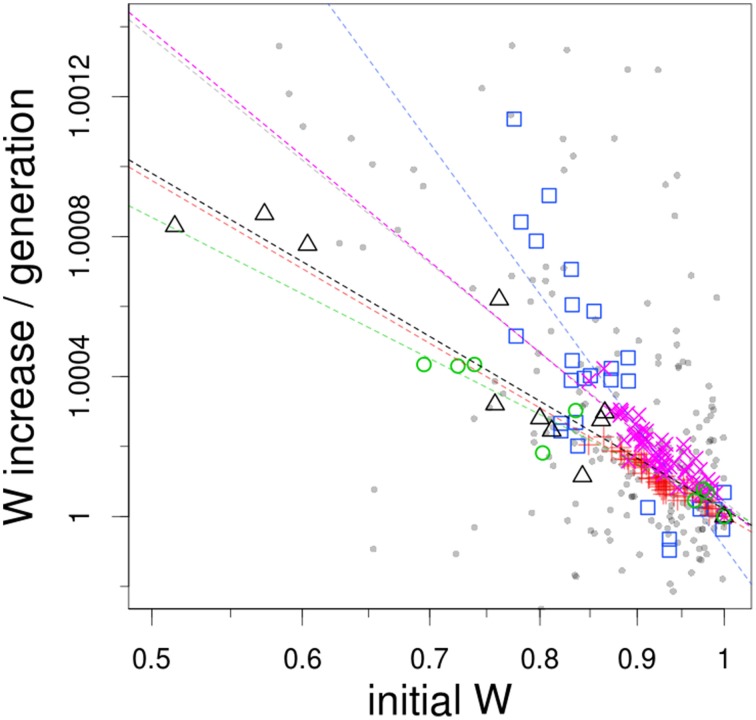
**Fitness increase as an inverse function of initial fitness**. The datasets used here correspond to evolution experiments with *S. cerevisiae* (BY4741 strain, gray small circles; DBY15108 strain at 250 generations, magenta crosses; at 500 generations, red diagonal crosses), *E. coli* (mutator MG1655 strain, blue squares; wild-type REL606 strain, green circles) and microvirid bacteriophages (black triangles). Dashed lines shows best-fit log-log linear regression model to each dataset (correlation coefficients: gray, 0.33; magenta, 0.80; red, 0.87; blue, 0.67; green, 0.92; black, 0.87; *F*-test, all *P* < 10–15). Note that both axes are on a natural logarithmic scale.

To allow for comparisons among datasets several standardization steps were required. First, fitness values from Barrick et al. (2010) and Rokyta's et al. (2009) datasets were converted to differences of Malthusian parameters, a metric compatible with classical population genetics (Chevin, 2011). Next, we estimated the extent to which an initial fitness difference between genotypes translates into a final fitness difference. This involved calculating the ratio of initial and final fitness of each genotype *i* against the most fit starting genotype of each dataset. The initial ratio was computed as *log*(*w_i_final_*/*w_max_start_*) whereas the ratio of fitness gains was calculated as *log*(*w_i_final_*/*w_i_start_*) – *log*(*w_max_final_*/*w_max_start_*). Finally, all values where divided by the number of generations elapsed in each experiment. This last step was required because the experiments differ in the number of generations elapsed (from 240 to 640), which has already been shown to alter the pattern of declining adaptability (Kryazhimskiy et al., 2014). The results of this analysis are showed in Figure 1 (data available in Supplementary Material).

In qualitative terms, low-fitness genotypes adapt markedly faster than high-fitness genotypes. More quantitatively, in all cases a log-linear relationship emerges between fitness gain and initial fitness, although correlation coefficients vary considerably (see legend of Figure 1). Szamecz's et al. (2014) and Perfeito's et al. (2014) data exhibit the worst fit to a log-linear model (R-squared of 0.33 and 0.67, respectively). In fact, Perfeito's et al. (2014) data is the only one that displays a better fit to an exponential regression, although only marginally (R-squared of 0.71). The noise of these two datasets is perhaps attributable to the presence of loss-of-function mutations, a consequence of the approach they followed in the assembly of their collection of genotypes. As discussed in Szamecz et al. (2014), for some loss-of-function mutations there may not exist simple ways to restore cellular physiology, so populations are *de facto* expelled from the domain of attraction of the original fitness peak. This suggests that the global picture of adaptation emerging from Figure 1 may be only applicable to populations relatively well-adapted to their environment, sharing comparable genetic potential.

The steepest slope of all the datasets is the one from Perfeito et al. (2014), which is probably due to the hypermutable nature of the strain employed in their experiment. Interestingly, a comparison of the regression lines from the two timepoints reported in Kryazhimskiy et al. (2014) suggests that the dependence of adaptability with initial fitness tend to vanish with evolutionary time. Finally, it is noteworthy that Rokyta's et al. (2009), Barrick's et al., (2010) and the latest of the Kryazhimskiy's et al., 2014) datasets exhibit such similarity in the slopes of the regression lines, despite coming from experiments with *E. coli*, microvirus and yeast respectively.

## What determines the rule of declining adaptability?

At first sight, it makes sense to think that worse-adapted genotypes exhibit larger fitness gains simply because they have more mutations at their disposal. That is, apart from having access to the same adaptive mutations as the reference strain, they can also substitute mutations that neutralize their initial fitness handicap. The problem is thus one of timescale. The pattern depicted in Figure 1 implies that low-fitness genotypes must fix both types of mutations in almost the same amount of time in which the reference strain fixes just one. But what could explain this difference on fixation rates?

One simple explanation, which was advanced by Moore et al. (2000), relates to population-genetic dynamics. Low-fitness populations should experience shorter waiting times between selective sweeps due to the higher availability of beneficial mutations. In addition, selective sweeps should be faster because beneficial mutations have stronger effects in poorly-adapted genotypes (Hermisson and Pennings, 2005). Things can get more complicated with the inclusion of clonal interference, but the general prediction is the same (Comeron et al., 2008): as a consequence of both phenomena, fitness gains per unit time should scale negatively with the fitness of the resident genotype.

While apparently straightforward, this dynamical explanation has some deep implications about the underlying fitness landscape. The argument relies on the strong assumption that the mutation rate and the average effect of beneficial mutations vary with genetic background. In other words, it implies that real fitness landscapes exhibit what can be termed as “macroscopic” epistasis (Good and Desai, 2015). It is important to note, however, that macroscopic epistasis can emerge in the absence of epistasis at the genetic level (‘microscopic’ epistasis). To illustrate this point, let us consider a genotype composed of a finite number of non-epistatic adaptive mutations, each one with a particular target size and a particular effect on fitness. In such scenario, the stronger and most common mutations will be substituted first (provided that small-effect mutations are not much more frequent). In consequence, some form of macroscopic epistasis will be observed as the distribution of fitness effects of fixed mutations change along adaptation. Indeed, it has been recently proven that, in general terms, any fitness trajectory can be recovered from such types of microepistasis-free landscapes (Frank, 2014; McCandlish et al., 2014; Good and Desai, 2015).

Yet, of course, the existence of microscopic epistasis is well-documented empirically (Elena and Lenski, 1997; de Visser and Hoekstra, 1998; Bull et al., 2000; Levin et al., 2000; Sanjuan et al., 2004; Weinreich et al., 2006; Chou et al., 2011; Khan et al., 2011; Schenk et al., 2013). Of particular interest is the observation of ‘diminishing returns’ epistasis: measurements on all the possible combinations among a small set of beneficial mutations showed that their fitness effects tend to diminish in more fit backgrounds (Weinreich et al., 2006; Chou et al., 2011; Khan et al., 2011). This behavior is perhaps intuitively explainable by the fact that there are physico-chemical limits to what is possible: diffusions of molecules, reaction's kintetics, and more complex processes cannot be instantaneous. So, all in all, it may seem that the pattern depicted in Figure 1 can ultimately emerge from biologically reasonable properties such as variation in the availability and effects of beneficial mutations, and biochemical and physical constraints.

## Toward a quantitative understanding of the patterns of declining adaptability

So far we have just identified some plausible mechanisms generative of macroscopic epistasis, which *per se* can only offer a qualitative explanation to the rule of declining adaptability. It still remains the problem of understanding quantitatively the log-log linear relationship shown in Figure 1. Indeed, it is truly remarkable to observe little if any degree of idiosyncrasy across systems as diverse as virus, bacteria and yeast. This similarity was completely unexpected, since at least two sources of heterogeneity could reasonably have affected the shape of the regression curves. On the one hand, datasets likely differ in their overall levels of adaptation to the particular experimental environment; that is, they start at different distances from their corresponding fitness peak. In addition, the genetic architecture of the traits under selection is expected to vary considerably, not only due to phylogenetic distance but also to the different strategies employed to construct each collection. We decided therefore to test the extent to which simple models could generate this pattern.

We performed computer simulations to explore four different models that can generate macroscopic epistasis (see Supplementary Material). The first model assumes that only the fitness effect of beneficial mutations diminishes with adaptation. This was implemented, inspired from Wiser et al. (2013), using an exponential distribution with parameter defined as α*_w_* = α_0_*w^g^*. As the fitness *w* increases, the mean effect of mutation—the inverse of α_*w*_—decreases. The second model considers that it is only the mutation rate, and not the effect, that changes. It involves therefore a constant distribution of effects (Silander et al., 2007) but a mutation rate that depends on the level of adaptation such as μ_*w*_ = μ_0_*w^−g^*. The third model is a simple finite sites model with multiplicative effect (McCandlish et al., 2014; Good and Desai, 2015), and is the only one that lacks microscopic epistasis. The fitness effects of beneficial mutations are drawn from an exponential distribution. The fourth model implements Fisher's Geometric Model (FGM) of adaptation (Fisher, 1930; Tenaillon, 2014), where the traits of an individual define a unique position in a multidimensional Euclidian space. There is just one optimal combination of trait values and fitness decays with the distance to that optimum. All models assumed an asexual population of size 10^6^ and a mutation rate of 10^−5^, ensuring therefore a clonal interference regime as is observed in most experimental settings.

**Figure 2:**
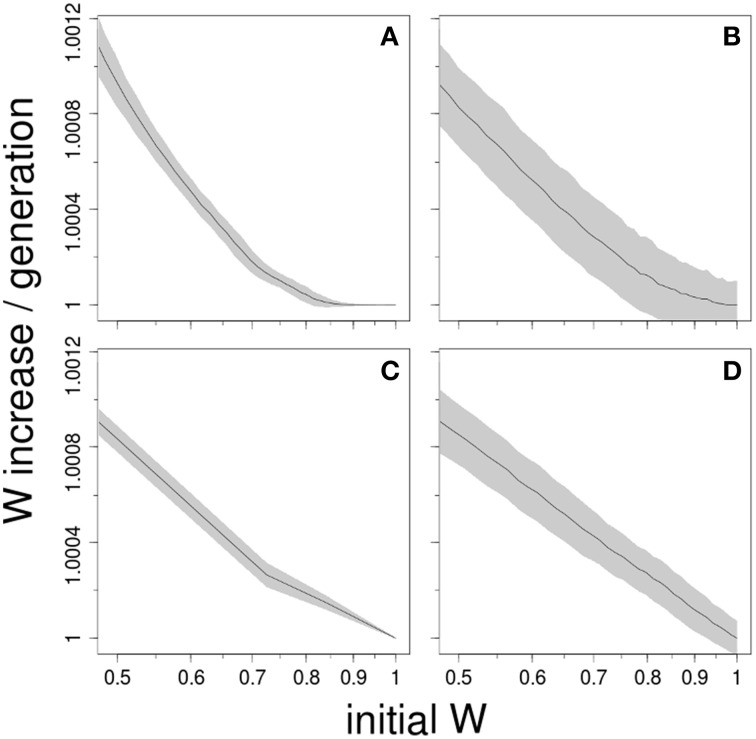
**Log-log-linear scaling is readily generated by many adaptation models**. The plots show the average ± SD of 1000 computer simulations of asexual populations adapting to a novel environment. All models explored here share the property of giving rise to macroscopic epistasis, albeit through completely different mechanisms: **(A)** only the average effect of beneficial mutations diminishes with adaptation; **(B)** only mutation rate declines with adaptation; **(C)** a finite-sites model; and **(D)** Fisher's Geometrical Model. Parameter range was chosen to ensure clonal interference dynamics.

Figure 2 shows that these very different models can give rise to log-log linear patterns. However, for the first three models, this type of relationship was only observed when the parameters lead to an unrealistic, massive fitness improvement (data not shown). This was unsurprising because under such scenario any model is expected to lead to a log-log-linear fit: initial fitness differences become negligible compared to the stochasticity of adaptation, so when they are subtracted to the final fitness, a linear relationship appears. When the rates of adaptation were moderate and similar to the experimental ones, these models were only able to produce a log-log linear fit for large initial fitness differences (see Figure 2). Roughly speaking, this simple analysis suggests that the rule of declining adaptability can emerge from a variety of adaptation models. Interestingly, the first three models require some parameter fine-tuning to recover the log-log-linear fit observed in Figure 1. In FGM, nonetheless, this log-log-linear fit is found consistently, and recovered numerically when mutations are rare, except from the closest vicinity of the fitness optimum (see Supplementary Material).

In the light of the models, the observed similarity of some of the regression curves is quite puzzling. That might suggest that not only the underlying model, but also the parameters can be conserved across systems. For instance, if the true model were some sort of finite-sites model, Figure 1 would mean that all these diverse model organisms exhibit about the same number of available beneficial mutations, with roughly the same distribution of target sizes and fitness effects. If it were FGM, it would imply that the number of phenotypic traits under selection, i.e., phenotypic complexity (Tenaillon et al., 2007) or the average mutation effects are somewhat comparable. However, the log-log linear fit is not the only prediction of FGM that is compatible with experimental observations (Tenaillon, 2014); these include: a negative gamma distribution of mutation effects (Martin and Lenormand, 2006), distributed pairwise epistasis with zero mean (Martin et al., 2007; Gros et al., 2009), diminishing-returns effect of beneficial mutations (Martin et al., 2007; Blanquart et al., 2014) or the potential existence of population size dependent fitness equilibrium (Silander et al., 2007). Moreover, these predictions are not restricted to beneficial mutations and can be extended to include discrete or continuous change of the optimum (Gordo and Campos, 2012; Bank et al., 2014; Matuszewski et al., 2014). Hence, FGM may offer an interesting perspective to investigate these dynamics more thoroughly.

## Concluding remarks

Here, we have re-analyzed published datasets from experimental microbial evolution to explore the empirical rule that the rate of adaptation is proportional to initial fitness. Such pattern implies some form of macroscopic epistasis; in other words, that the distribution of fitness effects of beneficial mutations varies along the adaptive process. Apart from the qualitative relationship between fitness gain and initial fitness, we found that this relationship fits a log-log-linear function; a feature displayed by all datasets despite their markedly different origins. We then used computer simulations to show that simple models implementing macroscopic epistasis can generate in some range this kind of behavior. Interestingly, Fisher's abstract geometric model appeared to generate quite robustly the observed pattern confirming that this model offers an interesting framework to investigate experimental evolution (Tenaillon, 2014).

An issue closely related to the topic discussed here concerns the nature of the fitness plateau that eventually emerges in long-term experimental evolution (Wiser et al., 2013; Good and Desai, 2015). Acording to Figure 1, if a log-log-linear relationship holds for all initial fitness differences, some fitness trajectory may be extrapolated. With a regression of absolute value β, fitness evolution with time *wt* can be computed as: *log*(*wt*/*w*0) = *log*(*w*1/*w*0) * (1 − (1 − β)*t*)/β, which suggests a fitness plateau of *log*(*w*1/*w*0)/β. However, how fast this plateau is reached and how high it might be can be significantly altered by the behavior of the regression close to 0 in Figure 1, a range were experimental resolution is limited. Hence, while some short-term prediction on the rate of evolution may be extracted from Figure 1, long-term evolution requires different experimental approaches.

In conclusion, and contrary to recent suggestions (Good and Desai, 2015), the universality of the patterns presented here suggest that the first steps of adaptation to novel conditions could perhaps be captured by a general theory of adaptation, rendering evolution statistically predictable at least at the macroscopic level. The construction of such a theory will benefit from further effort to extend evolution experiments with strains with different starting fitness to a larger fitness range, higher fitness accuracy and increased replication.

### Conflict of interest statement

The authors declare that the research was conducted in the absence of any commercial or financial relationships that could be construed as a potential conflict of interest.
