# Supplementary material for: The rule of declining adaptability in microbial evolution experiments
Source: Front Genet. 2015 Mar 11;6:99. doi: 10.3389/fgene.2015.00099 (PMC4356158; doi:10.3389/fgene.2015.00099)
Supplement: Supplementary file 1 [file Presentation_1.PDF]

Simulation details:

Simulations were performed with two independent architectures. First, they were performed with an individual-based model as described previously (Tenaillon *et al*. 1999). Basically, following mutation and selection, individuals were randomly sampled to generate genetic drift. Second, to run faster, a semi-deterministic model was build. In that model, selection is followed by the random production of mutations, whose survival probability a drawn immediately according to their fitness effect with probability $2s$. This version which does not allow the fixation of deleterious mutations and limits the diversity of beneficial mutation to contending mutations (mutations surviving drift) runs much faster than the individual-based model and is accurate as long as N is large and μ is small.

In the two architectures, the genotypes are encoded in the same way: for model 1 and 2, only fitness is stored as it is sufficient to define the distribution of beneficial mutations. Both mutation rate and fitness effects of new mutations are drawn from an exponential distribution (see main text). In model 2 simulations started in a clonal interference mutation regime (i.e.,large $N\mu$). However, as fitness increased the mutation rate decreased, such that at some point $N\mu << 1$ and the weak mutation regime described below applied.

For the finite-sites model, (Model 3) we varied the number of bi-allelic loci from 30 to 150. The ancestral clone only consisted of wild-type alleles (denoted '0') and had a fitness of 1. Mutations randomly created mutant alleles (denoted '1') with fitness-effects drawn from an exponential distribution.

For model 4, Fisher's Geometric model, a phenotypic landscape of n dimensions (2 to 30 have been tested) is used. The encoded genotype is the coordinates in that space, and fitness is defined by

$W(d)=e^{-d^2}$ where d is distance to the optimum. A mutation is a multivariate Gaussian deviation from the focal position in the space. The mean effect of mutations has been set to *1%*, such that the standard deviation of the Gaussian deviations along each axes is $\sigma=\sqrt{0.02/n}$. If the fitness decay is not quadratic and fitness is of the form $W(d)=e^{-d^Q}$ with *Q* different of *2*, the log-log-linearity is not anymore observed. However, the distributions of epistatic interactions in several experimental evolution systems are compatible with *Q=2* (Gros *et al*. 2009, Martin *et al*. 2007).

Analytical and numerical results

In the main text we showed results from simulations under clonal interference. For the sake of completeness, we present here some analytical and numerical results in the weak mutation regime. In that regime mutation rate is so small that mutations are lost or reach fixation independently. Out of the 4 models we present, all but the Finite Sites model (Model 3) have a distribution of fitness effects that has a pure dependency on fitness. For each of these models, we can compute numerical solutions showing the dependency of the final log-fitness difference on the initial log-fitness difference as illustrated in Figure 1. Basically, we can compute the increase in fitness through time as:

$$\frac{dW}{dt}=WN\mu\int_0^\infty 2s^2\,p(s)\,ds \qquad (1)$$

with *N* being the population size, *μ* the mutation rate, and *p(s)* the distribution of the fitness effects of beneficial mutations. We use *2s* as that probability of fixation, assuming *N>>1*.

Change in fitness effects (Model 1) and mutation rate (Model 2):

In both models, the distribution of beneficial mutations being exponential with parameter *α*, we have:

$$\frac{dW}{dt}=2\mathrm{WN}\mu\frac{1}{\alpha^2} \tag{2}$$

In model 2, $\mu(W)=\mu_0 W^{-g}$ can be substituted in the previous equation to find:

$$W(t,W_0,g,\alpha)=\left(g\frac{2\mathrm{N}\mu}{\alpha^2}t+W_0{}^g\right)^{1/g} \tag{3}$$

From that equation, we can create a plot equivalent to the one presented in Figure 1. We first need a population of maximum fitness to be used as a reference. Given fitness W_{i_max}, we can compute:

-(i) the time *T* at which it will be reached if a population was initiated from a fitness of W_0, such that $W_{i\_max}=W(T)$

-(ii) the fitness it will reach, *W(T+dt)* after *dt* mutations of adaptation.

For all $t<T$, we can then compute the *y* and *x* component of the parametric plot presented in Figure S1. On the *x* axis we use:

$$log\left(\frac{W(t)}{W(T)}\right) \tag{4}$$

and on the *y* axis :

$$\log\left(\frac{W(t+dt)W(T)}{W(t)W(T+dt)}\right) \qquad (5)$$

Independent of the set of parameters, if the log-fitness differences on the x axis are comparable to the one of Figure 1, a curvature is observed as in Figure S1. This plot was obtained numerically in the weak mutation limit with parameters (*W0=1, g=4,* $2\mathrm{N}\,\mu_0=0.00002\,\alpha^2$ *, T=100000, dt= 500*). In the case of model 1, it is not the mutation rate but the parameter of the exponential that changes according to fitness: $\alpha=\alpha_0 W^g$. The results are identical to the previous case with *2g* instead of *g*.

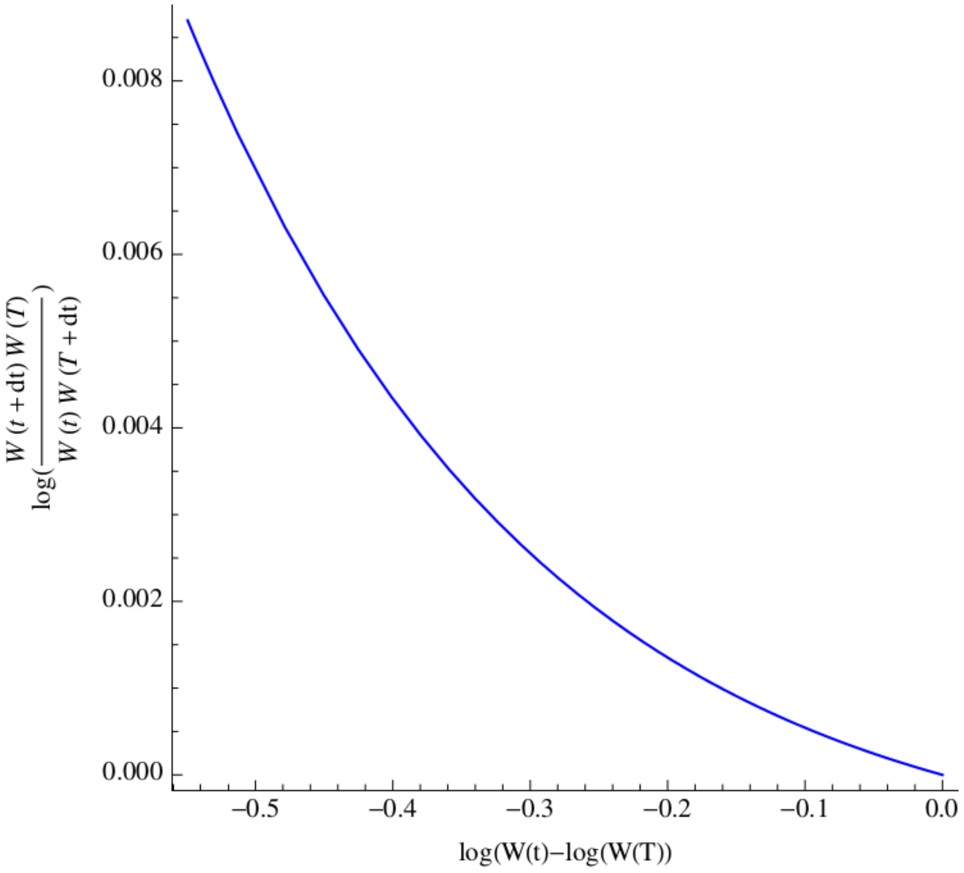

Figure S1: Difference in log fitness improvement as a function of the initial differences in model 2 characterized by a decrease in beneficial mutation rate as fitness increases.

Alternatively, the dependency of log-fitness increase on initial log-fitness difference can be observed by computing the derivative of the rate of log-fitness increase with respect to log-fitness. If we write (for model 2 and see Figure S2):

$$R(\log(W)) = \frac{d\log(W)}{dt} = \frac{2\mathrm{N}\,\mu_0}{\alpha^2} e^{-g\log(W)} \quad (6)$$

we are interested in:

$$\frac{dR(\log(W))}{d\log(W)} = -g\frac{2\mathrm{N}\,\mu_0}{\alpha^2} e^{-g\log(W)} \quad (7)$$

This derivative is always negative and is a decreasing function of *W*. In other words when *W* is small, there is an important effect of initial fitness difference, which is less marked when fitness is high.

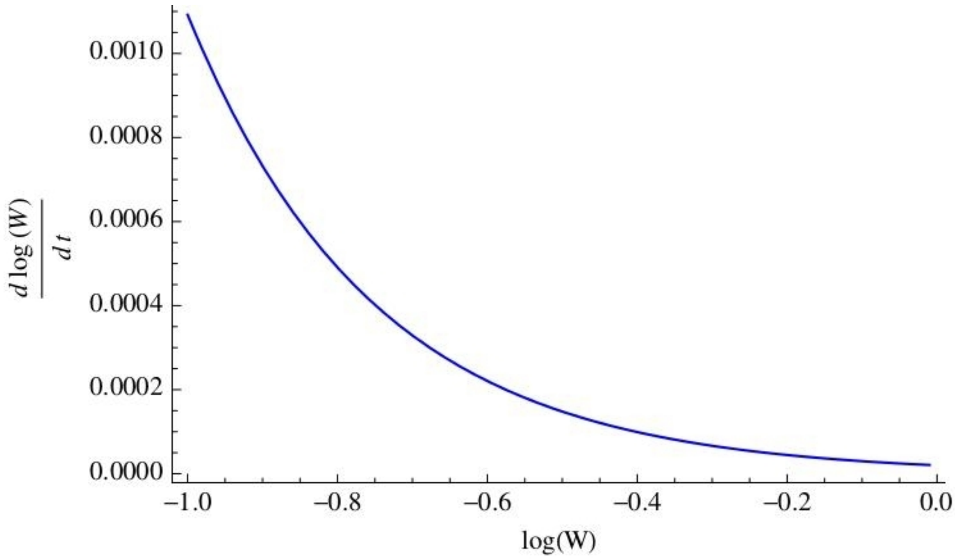

Figure S2: Rate of log-fitness improvement with time as a function of log-fitness in model 2 (*g=4,* $2\mathrm{N}\,\mu_0 = 0.00002\,\alpha^2$)

Fisher's Geometrical Model (Model 4)

In Fisher model, the equations are more complex and we rely more heavily on numerical solutions. In a model with *n* dimensions, with fitness of the form $w(d)=e^{\frac{-d^Q}{\alpha}}$, *d* being the distance to the optimum and *Q* an epistasis parameter, isotropic mutations resulting from a multivariate Gaussian of standard deviation *σ*, the distribution of mutations effects on log-fitness has a closed formula (Tenaillon, 2014):

$$p(s)=\frac{e^{-\frac{\left(\frac{-s+s0}{\alpha}\right)^{2/Q}+\left(\frac{s0}{\alpha}\right)^{2/Q}}{\sigma^2}}}{\alpha Q\sigma^2}\left(\frac{-s+s0}{\alpha}\right)^{\frac{\frac{n}{2}+1-Q}{Q}}\left(\frac{s0}{\alpha}\right)^{\left(1-\frac{n}{2}\right)/Q} I_{\frac{n}{2}-1}\left[\frac{1}{\sigma^2}\left(\frac{-s+s0}{\alpha}\right)^{1/Q}\left(\frac{s0}{\alpha}\right)^{1/Q}\right] \quad (8)$$

in which *s0* is the maladaptation of the strain $s0=-\log(W_0)$, $I_k(y)$ is the modified Bessel function of the first kind. However such formula cannot be integrated over *s* (i.e., all beneficial mutations). Nevertheless, if *Q=2* and $\alpha=1/2$ and *n* large, the distribution is well approximated by a normal distribution:

$$p(s)\approx N\left(\frac{-\sigma^2 n}{2},\sqrt{\sigma^2\left(2s0+\frac{\sigma^2 n}{2}\right)}\right)=N\left(\bar{s},\sqrt{\sigma^2(2s0-\bar{s})}\right) \quad (9)$$

In Figure S3, we plot the rate of log-fitness increase with time as a function of the initial log-fitness:

$$R(\log(W))=\frac{d\log(W)}{dt} \quad (10)$$

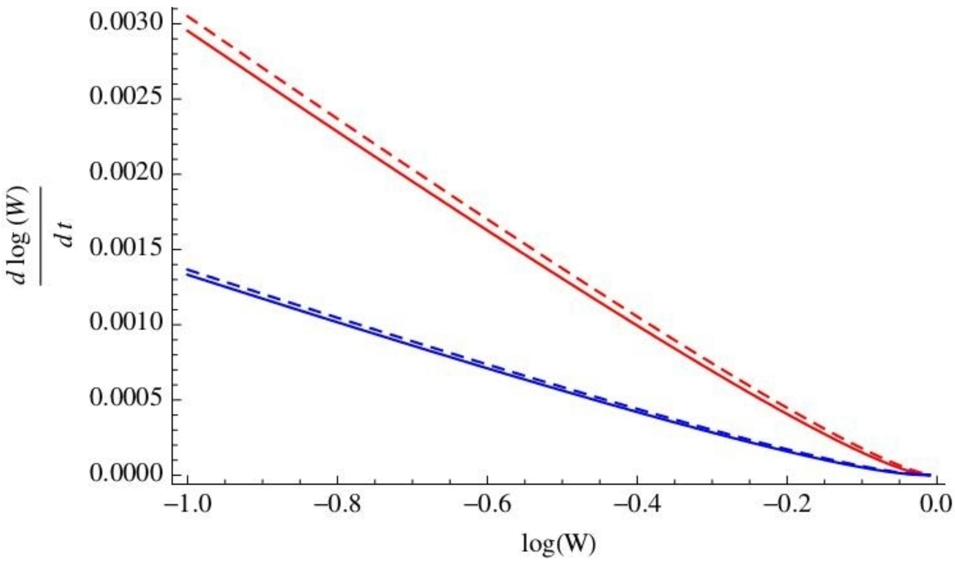

Figure S3: The derivative of log-fitness with time is presented as a function of log-fitness. Dashed lines represent the Gaussian approximation, and solid lines the numerical approximation of the exact distribution of mutation effect. Parameters are *Nµ=1,* $\sigma=0.01$ *, n=20* (blue) or *n=10* (red).

Figure S3 shows that our approximations match our simulation results very well and supports the conclusions of the simulations presented in the main text, suggesting a linear relationship between log-fitness improvement and log-fitness. To go further we can compute:

$$\frac{d\,R(\log(W))}{d\log(W)}=-2\mathrm{N}\mu\,\sigma^2\,Erfc\left(\frac{n}{2}\sqrt{\frac{\sigma^2}{-4\log(W)+n\sigma^2}}\right)=4\,N\mu\frac{\bar{s}}{n}\,Erfc\left(\sqrt{\frac{-n\bar{s}}{-8\log(W)-\bar{s}}}\right) \quad (11)$$

in which $\bar{s}$ is the mean log fitness effect of mutations and Erfc(x) is the complementary error function that converges to 1 when x goes to 0. This function converges to $4\mathrm{N}\mu\frac{\bar{s}}{n}$ and lead therefore to a linear behavior as observed in Figure 1. The convergence is slow if the product $n\bar{s}$ is large.

Nevertheless, so far most quantitative estimates of both terms suggest the product is rather small: with $n < 30$ and $\bar{s} < 0.1$.

References

S1. Gros PA, Le Nagard H, Tenaillon O. 2009. The evolution of epistasis and its links with genetic robustness, complexity and drift in a phenotypic model of adaptation. *Genetics*. 182(1):277–93

S2. Martin G, Elena SF, Lenormand T. 2007. Distributions of epistasis in microbes fit predictions from a fitness landscape model. *Nat. Genet.* 39(4):555–60

S3. Tenaillon, O., 2014. The utility of Fisher's geometric model in evolutionary genetics. Annu. Rev. Ecol. Evol. Syst. 45, 179-201

S4. Tenaillon O, Toupance B, Le Nagard H, Taddei F, Godelle B. 1999. Mutators, population size, adaptive landscape and the adaptation of asexual populations of bacteria. *Genetics*. 152(2):485–93.
